# A missing link for efflux pumps

**DOI:** 10.7554/eLife.111230

**Published:** 2026-03-25

**Authors:** Hilal Wehbi, Isabelle Broutin

**Affiliations:** 1 Laboratoire CiTCoM, Faculté de Santé, Université Paris Cité and CNRS Paris France

**Keywords:** efflux pump, MacAB-TolC, AcrABZ-TolC, type I secretion, lipoprotein, membrane protein, *E. coli*

## Abstract

A lipoprotein called YbjP could be the answer to a puzzle about efflux pumps in gram-negative bacteria.

**Related research article** Horne J, Kaplan E, Jin BHS, Abbot K, Flores V, Petsolari E, Gradon JM, Ntsogo Y, Harris A, Yu D, Zarkan A, Luisi BF. 2026. A lipoprotein partner for the *Escherichia coli* outer membrane protein TolC. *eLife* **15**:RP110666. doi: 10.7554/eLife.110666.

We have become used to treating bacterial infections with antibiotics, but in recent decades there has been an increase in the number of bacterial strains for which there is no efficient treatment. Nosocomial bacterial infections – infections acquired in hospitals – are especially concerning (Sati et al., 2025).

Bacteria have developed various different strategies to resist antibiotics (Darby et al., 2023), including the use of ‘efflux pumps’ to expel the drugs from cells before they can reach their target. Gram-negative bacteria such as *E. coli* possess two protective membranes, so an efflux pump has to be able to transport the antibiotic through both membranes and the space between them.

Several families of efflux pumps have been discovered in gram-negative bacteria. These pumps are formed of three components, each made of different proteins. In general, one component is inserted in the outer membrane of the bacteria, one is inserted in the inner membrane, and the third connects the other two (Figure 1). However, while there are many similarities between various efflux pumps, there are also important differences: for instance, some rely on ATP as an energy source to transport the antibiotic being expelled, whereas others exploit the proton motive force (Li et al., 2015; Alav et al., 2021).

**Figure 1.**
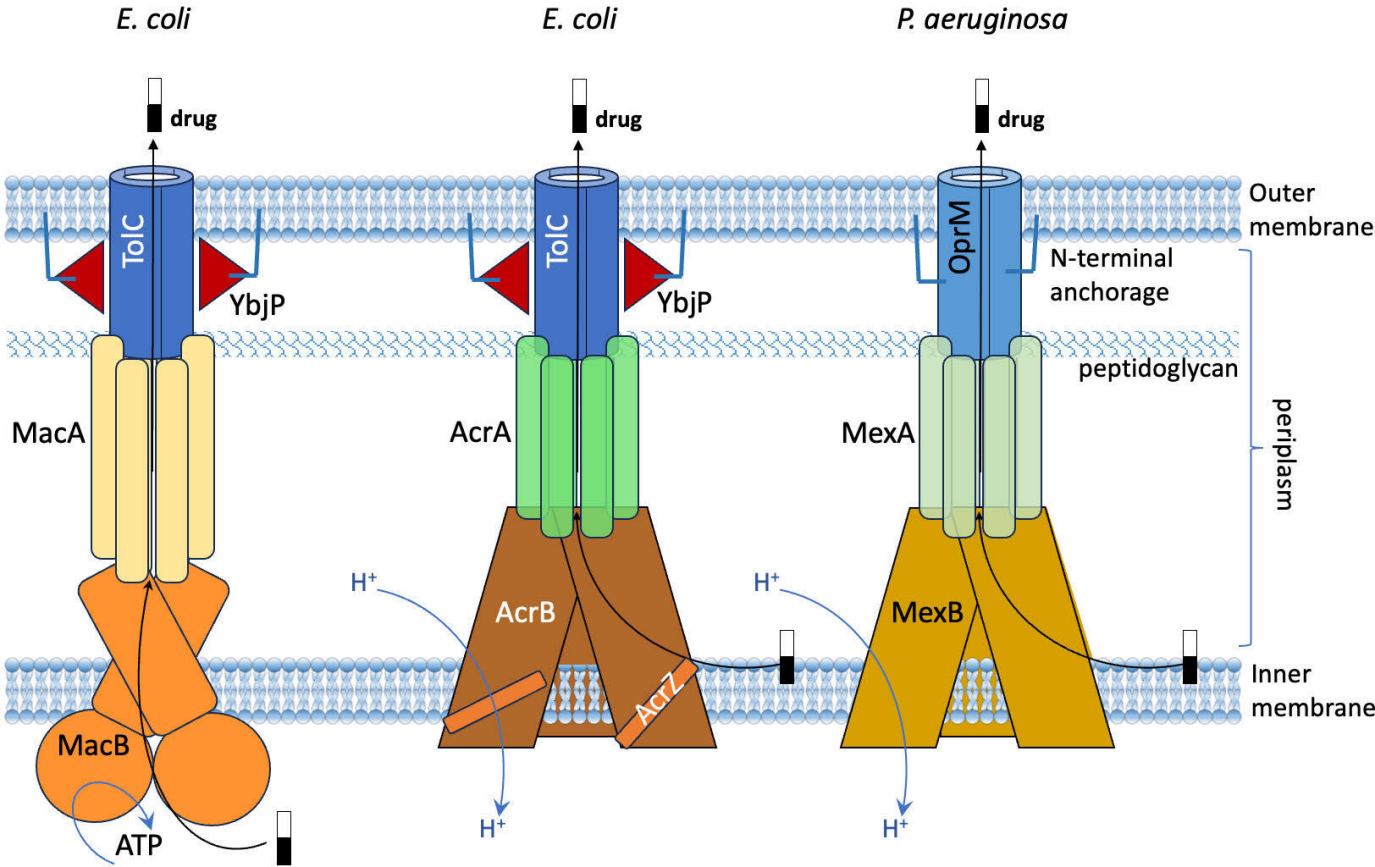
Comparison of three efflux pumps that can expel drugs from gram-negative bacteria. Schematic presentations of efflux pumps found in *E. coli* (MacAB-TolC; left: AcrABZ-TolC; middle) and *P. aeruginosa* (MexAB-OprM; right). In gram-negative bacteria, the membrane is formed of an inner membrane (bottom), an outer membrane (top), and a layer of peptidoglycan in the space between these two membranes (which is called the periplasm). In MacAB-TolC, the MacB proteins embedded in the inner membrane rely on ATP as a source of energy. In both AcrABZ-TolC and MexAB-OprM, the proteins embedded in the inner membrane rely on the proton motive force for energy. In MexAB-OprM, the OprM protein is anchored in the outer membrane (top) by an N-terminal extension (blue line), but the TolC protein in the *E. coli* efflux pumps does not have such an extension. However, researchers have discovered the presence of a lipoprotein (YbjP; red triangles) with a similar extension (blue line) close to TolC.

To develop new drugs that target these efflux pumps, it is important to understand how they work at the molecular level, so it is necessary to solve their three-dimensional structure. Now, in eLife, Ben Luisi and colleagues at the University of Cambridge – including Jim Horne and Elise Kaplan as joint first authors – report new insights into two efflux pumps found in *E. coli* (Horne et al., 2026). In the MacAB-TolC pump, a dimer made of two MacB proteins in the inner membrane is connected to a trimer of TolC proteins in the outer membrane by a hexamer of MacA proteins (Fitzpatrick et al., 2017). In the AcrABZ-TolC pump, a trimer of AcrBZ proteins in the inner membrane is connected to a trimer of TolC proteins in the outer membrane by a hexamer of AcrA proteins (Wang et al., 2017).

Horne et al. used a technique called cryo-electron microscopy to determine the structure of the efflux pumps. This typically involves producing the proteins in large quantity, extracting them from the bacterial cell, and then stabilizing them through the use of detergents that mimic the lipids found in biological membranes. However, by using peptidiscs (Carlson et al., 2018) rather than detergents to stabilize MacAB-TolC, Horne et al. were able to see evidence for an extra protein near the TolC in the outer membrane. The resolution was sufficient to interpret the images and build an incomplete protein skeleton that could be compared to a database of protein models. This allowed the extra protein to be identified as a lipoprotein called YbjP. The researchers also verified that purified YbjP is able to interact with TolC. Horne et al. then went on to solve the structure of two *E. coli* efflux pumps (MacAB-TolC and AcrABZ-TolC) in the presence of YbjP, and found that this did not modify the structure of either pump.

Independently, in a separate eLife paper, Xiaofei Ge, Zhiwei Gu and Jiawei Wang of Tsinghua University also identified the presence of YbjP in the outer membrane of *E. coli* and solved the structure of AcrABZ-TolC-YbjP (Ge et al., 2025), showing that research evolves in the same way everywhere when knowledge is shared.

The discovery of YbjP in the outer membrane near TolC could help explain something that has been puzzling researchers in this field. In many efflux pumps, the protein embedded in the outer membrane has an extension that anchors it in the membrane. One example is the MexAB-OprM efflux pump in the pathogenic bacteria *P. aeruginosa* (Figure 1; Tsutsumi et al., 2019; Glavier et al., 2020). TolC does not have such an extension, but YbjP does, and since it can interact with TolC, YbjP may have a role in anchoring TolC in the outer membrane.

Genetic analyses by Horne et al. also revealed that YbjP is not always present with TolC in other organisms. To explore further, the researchers deleted the genes for TolC and/or YbjP from the *E. coli* genome. Surprisingly, they found that bacteria lacking YbjP were still able to survive under classical culture conditions, even in the presence of antibiotics. This means that YbjP is not essential for the basic pumping activity of the AcrABZ-TolC system. Nevertheless, additional analyses of protein expression suggested that the absence of YbjP slightly affects how bacteria respond to environmental conditions.

These results suggest that YbjP might help bacteria adapt to environmental challenges, such as changes in nutrient availability or the presence of toxins: in this context YbjP might, for example, escort TolC while it is being inserted into the outer membrane. While YbjP is not essential for basic function of efflux pumps, it likely helps an important component of these pumps – the TolC protein – to perform optimally under stress. Therefore, in addition to deepening our understanding of bacterial survival mechanisms, the latest findings could also open new doors for future research into how we might exploit these systems to fight infections.
